# Superior mesenteric arteriovenous fistula mimicking abdominal aortic aneurysm, treated with a single vascular plug

**DOI:** 10.1016/j.jvscit.2026.102217

**Published:** 2026-03-02

**Authors:** Ihza Fachriza, Akhmadu Muradi, David Christianta, Firman Syah, Farhan Alief Waluyo, Bintang Heiza Yudistira

**Affiliations:** a Faculty of Medicine, Universitas Indonesia, Jakarta, Indonesia; b Division of Vascular and Endovascular Surgery, Department of Surgery, Cipto Mangunkusumo National General Hospital, Jakarta, Indonesia

**Keywords:** Arteriovenous fistula, Case report, Embolization, Superior mesenteric artery, Vascular plug

## Abstract

Superior mesenteric arteriovenous fistula (SMAVF) is a rare postoperative vascular abnormality that can mimic more common causes of pulsatile abdominal masses. We report the case of a 49-year-old man with a history of laparotomy for volvulus 20 years earlier who presented with a pulsatile epigastric mass. Computed tomography angiography identified a 10 × 12 mm SMAVF, confirmed on angiography. Endovascular closure using a single Amplatzer vascular plug achieved complete occlusion without complications. This case underscores the importance of considering SMAVF in patients with atypical abdominal pulsations and highlights the effectiveness of a minimally invasive, single-device endovascular approach.

Superior mesenteric arteriovenous fistula (SMAVF) is a rare vascular abnormality characterized by an abnormal connection between the superior mesenteric artery (SMA) and the mesenteric or portal vein. An SMAVF most commonly arises as an iatrogenic complication after abdominal surgery, especially bowel resection or mesenteric vessel manipulation, but may also result from abdominal trauma, penetrating injury, inflammatory processes, or, rarely, congenital vascular anomalies. First reported by Movitz and Finne in 1960, SMAVF has an incidence of approximately 0.09% and is associated with high mortality rates ranging from 39% to 77%, predominantly affecting young males.^1^ A review of the past two decades shows that only a small number of SMAVF cases have been published, mostly as isolated reports or small series, underscoring the rarity of this condition. Additional case reports are therefore valuable for broadening understanding of its diverse presentations and treatment options, including the effectiveness of modern endovascular techniques such as single vascular plug embolization demonstrated in this case. The patient provided written informed consent for the publication of all clinical details and accompanying images included in this case report.

## Case report

**Fig 1:**
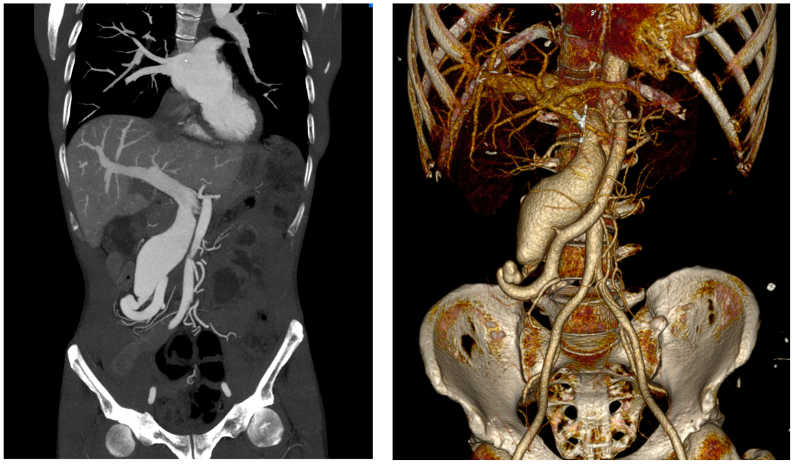
Computed tomography angiography findings. (Left) Two-dimensional coronal image showing dilatation of vascular lesion enlargement to the hepatic vasculature. (Right) Three-dimensional image showing the superior mesenteric artery (SMA) and dilated superior mesenteric vein (SMV) connected by a fistula.

**Fig 2:**
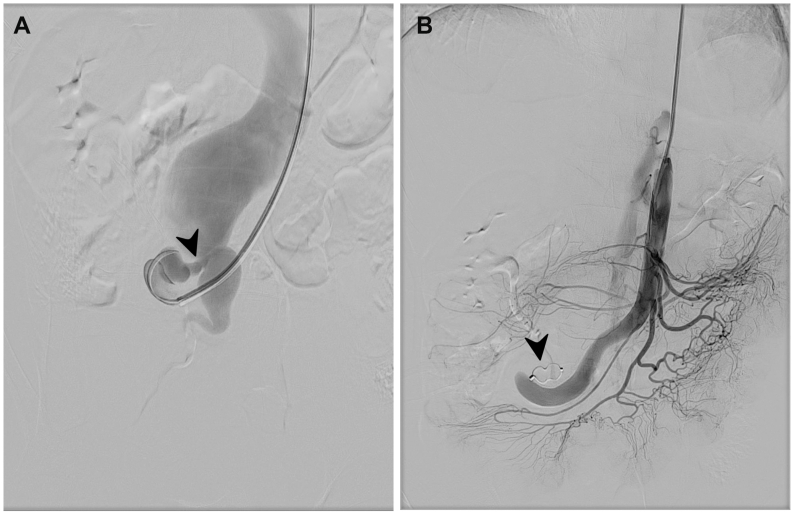
Pre-embolization and post embolization arteriography.**(A)** Pre-embolization arteriography from superior mesenteric artery (SMA), shows fistula (*black arrowhead*) to the dilated superior mesenteric vein (SMV. **(B)** Postembolization arteriography, shows complete closure of the fistula (*black arrowhead*).

A 49-year-old man presented with pulsatile mass on the epigastric region over 6 months. No gastrointestinal tract complaint accompanied the symptoms or any other diseases. The patient had an unremarkable medical, family, and psychosocial history, except for a laparotomy performed 20 years earlier for ileocolic resection and anastomosis owing to volvulus. Physical examination revealed a palpable pulsatile mass with an associated thrill in the epigastric region. Laboratory findings were unremarkable. Owing to suspicion of abdominal aortic aneurysms, computed tomography angiography and arteriography was performed and revealed a SMAVF with clearly delineated arterial and venous components (Figs 1 and 2, *A* Supplementary Video 1, online only).

The procedure began with left brachial artery access using a 6F sheath. An 0.035-inch, 260 cm soft wire and a 5F Berenstein diagnostic catheter (Merit) were used to cannulate the SMA, followed by SMA arteriography that identified a 10 × 12 mm AVF at the mesenteric artery-vein junction. The fistula was crossed with a Bern catheter, after which the wire was exchanged for an Amplatz stiff wire to improve support for device navigation. The sheath was then replaced with a 6F, 90 cm DuraSheath (CMI) positioned at the target segment, and a 15-mm Amplatzer Vascular Plug II (Abbott Vascular) was deployed to achieve approximately 50% oversizing relative to the target vessel, within a landing zone corresponding to the fistulous segment that, based on angiographic assessment, showed no residual SMA branch occlusion. Postprocedural arteriography confirmed complete closure, and no additional interventions were required (Fig 2, *B*, Supplementary Video 2, online only). Clinically, the pulsatile epigastric mass resolved, and laboratory results remained normal. At 1 month, in-person follow-up was not feasible; the patient remained asymptomatic, with planned computed tomography angiography, cardiac, and hepatic evaluation. The lack of early imaging is acknowledged as a limitation.

## Discussion

SMAVF is commonly found in male patients, particularly those >40 years old, with a history of abdominal surgery, such as bowel resections or trauma.^2^ This case aligns with epidemiological findings, with a 49-year-old male patient having a laparotomy 20 years ago owing to volvulus. Like many cases, SMAVF can develop years after surgery, which increases the risk of vascular complications like AVFs.

The most common symptoms of SMAVF include abdominal pain after meals, often accompanied by diarrhea, owing to abnormal blood flow causing ischemia in the intestines.^3^^,^^4^ In this patient, the only clinical sign was a pulsating mass in the epigastric region, without other gastrointestinal symptoms like bleeding or diarrhea. This could be due to the relatively low blood flow in his fistula. A low-flow fistula does not affect the systemic circulation enough to cause more severe complications like gastrointestinal bleeding.^5^

The pulsating mass felt by the patient arose from abnormal shunting of blood from the SMA to the vein, leading to the enlargement of the mesenteric vessels and turbulent blood flow, which creates a palpable, pulsating sensation in the abdomen.^6^^,^^7^ Given the patient's remote surgical history, it is likely that the fistula developed silently over time, with symptoms emerging only many years after laparotomy.^3^^,^^7^ In this case, no systemic manifestations were identified, consistent with a Schobinger stage II AVF.

The management of SMAVF includes surgical and endovascular approaches. Surgical resection or ligation, with or without bypass, may be required for high-flow or large-vessel fistulas, but is technically challenging and associated with reported mortality rates of ≤18%. Endovascular therapy is, therefore, preferred and includes coil embolization, covered stent placement, and vascular plug deployment. Although coils and stents may be limited by device burden and anatomical constraints, vascular plugs are well-suited for direct, well-defined fistulas with an adequate landing zone, supporting morphology-guided treatment selection.^6^^,^^8^^,^^9^

**Fig 3:**
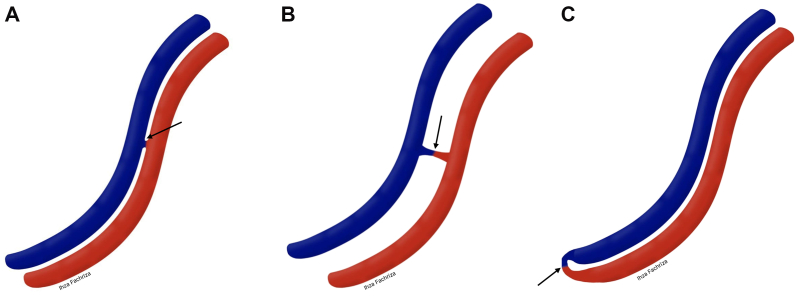
Schematic illustration of Superior mesenteric arteriovenous fistula (SMAVF) morphology. **(A)** Type 1A, a side-to-side fistula without a discrete fistulous segment. **(B)** Type 1B, a side-to-side fistula with an identifiable fistulous segment. **(C)** Type 2, an end-to-end fistula. *Red* represents the artery, *blue* represents the vein, and *arrowheads* indicate the fistula.

Based on the authors' review of previous case reports, the morphology of SMAVF can be classified into three types: type 1A SMAVF, characterized by a side-to-side fistula without a defined fistulous segment (Fig 3, *A*); type 1B SMAVF, defined as a side-to-side fistula with an identifiable fistulous segment (Fig 3, *B*); and type 2 SMAVF, which presents as an end-to-end fistula (Fig 3, *C*).^10^ Type 1A SMAVFs are best treated with a covered stent to preserve distal perfusion.^10^ In type 1B SMAVFs, fistula closure may be achieved using a covered stent, coils, or a vascular plug. For type 2 SMAVFs, closure with a vascular plug is considered more appropriate.^10^ When planning endovascular closure, it is crucial to assess the fistula's orientation, angulation, diameter, and its relationship to nearby mesenteric branches. Incorrect device sizing or positioning can result in incomplete occlusion, device migration, or inadvertent blockage of essential mesenteric vessels.

In the present case, angiography demonstrated a type 2 SMAVF, presenting as an end-to-end fistula between the SMA and SMV, making it amenable to vascular plug occlusion. Consistent with prior case reports, treatment selection was based on fistula morphology, diameter, landing zone, and preservation of distal mesenteric branches, rather than classification alone.

Although coils, covered stents, and vascular plugs have been used for SMAVF closure, this case underscores the value of morphology-guided device selection. Coils and stents may be limited by device burden, vessel tortuosity, and risk to adjacent branches, whereas a direct type 2 SMAVF is well-suited to treatment with a single vascular plug.^1^^,^^6^ Angiography showed a end-to-end fistula, with no distal SMA branches at the landing zone and a diameter of 10 × 12 mm, supporting stable plug deployment with preservation of mesenteric perfusion.

In this case, the prognosis was favorable because the condition was identified before the development of systemic complications related to a high-flow fistula and imaging was readily available, allowing timely endovascular treatment and complete symptom resolution. Although no diagnostic challenges were encountered, visceral arteriovenous malformations including SMAVF are often under-recognized and may be mistaken for more common conditions such as abdominal aortic aneurysm. Endovascular techniques such as coil or plug embolization or covered stent placement have been reported as effective treatment options, with rapid symptom improvement and a low incidence of serious complications.

The main strength of this case report is its demonstration that single vascular plug embolization is an effective and safe minimally invasive treatment for simple, direct SMAVF. This technique provides a lower morbidity alternative to coil embolization, stenting, or surgical repair, an especially important advantage for patients with complex surgical histories. The successful occlusion using only one device, without immediate complications, highlights the technical efficiency of this method. Additionally, the case emphasizes the rarity of SMAVF and its potential to mimic an abdominal aortic aneurysm, underscoring the need for clinicians to consider atypical causes of pulsatile abdominal masses.

Despite these strengths, this report is limited by its single-case design, restricting generalizability given the anatomical variability of AVFs. The lack of long-term follow-up precludes assessment of recurrence or delayed complications, and limited comparison with alternative embolization techniques prevents definitive conclusions regarding the superiority of a single vascular plug approach.

## Conclusions

A 49-year-old man with a history of ileocolic laparotomy was diagnosed with SMAVF and successfully treated with a single vascular plug. SMAVF is a rare but potentially fatal complication, and delayed diagnosis may lead to life-threatening bleeding. Clinical manifestations can be highly variable, emphasizing the importance of accurate diagnosis and prioritizing emergent conditions first. Computed tomography angiography remains crucial for diagnosis and anatomical delineation, whereas careful evaluation of fistula characteristics guides appropriate treatment selection. Given the diverse presentations and association with prior abdominal surgery, SMAVFs should be considered in the diagnostic workup of patients with unexplained pulsatile abdominal masses, and future guidelines may benefit from incorporating these considerations.

## Funding

None.

## Disclosures

None.

## Appendix (online only)

Supplementary Video 1 Pre-intervention angiography revealing a superior mesenteric arteriovenous fistula (SMAVF), characterized by an early-filling, high-flow communication between the superior mesenteric artery (SMA) and an enlarged draining venous segment, with a clearly recognizable border between the arterial and venous components.

Supplementary Video 2 Postintervention angiography demonstrating complete closure of the superior mesenteric arteriovenous fistula (SMAVF) after vascular plug deployment.
